# Pathological evidence for residual SARS-CoV-2 in pulmonary tissues of a ready-for-discharge patient

**DOI:** 10.1038/s41422-020-0318-5

**Published:** 2020-04-28

**Authors:** Xiao-Hong Yao, Zhi-Cheng He, Ting-Yuan Li, Hua-Rong Zhang, Yan Wang, Huaming Mou, Qiaonan Guo, Shi-Cang Yu, Yanqing Ding, Xindong Liu, Yi-Fang Ping, Xiu-Wu Bian

**Affiliations:** 1 Institute of Pathology & Southwest Cancer Center, Southwest Hospital, Third Military Medical University (Army Medical University), Chongqing, 400038 China; 2 Key Laboratory of Tumor Immunopathology, Ministry of Education of China, Chongqing, 400038 China; 3 Department of Vascular Surgery, Southwest Hospital, Third Military Medical University (Army Medical University), Chongqing, 400038 China; 4 Department of Cardiovascular Diseases, Chongqing Three Gorges Central Hospital, Chongqing, 404000 China; 5 Department of Pathology, Xinqiao Hospital, Third Military Medical University (Army Medical University), Chongqing, 400038 China; 6 Department of Stem Cell and Regenerative Medicine, Southwest Hospital, Third Military Medical University (Army Medical University), Chongqing, 400038 China; 7 Department of Pathology, Nan Fang Hospital, Southern Medical University, Guangzhou, 510515 China

**Keywords:** Mechanisms of disease, Pattern recognition receptors

**Dear Editor,**

SARS-CoV-2, a novel coronavirus and causing COVID-19, has given rise to a worldwide pandemic.^1,2^ So far, tens of thousands of COVID-19 patients have been clinically cured and discharged, but multiple COVID-19 cases showed SARS-CoV-2 positive again  in discharged patients,^3^ which raises an attention for the discharged patients. Also, there is an urgent need to understand the pathogenesis of SARS-CoV-2 infection. Here, we conducted postmortem pathologic study in a ready-for-discharge COVID-19 patient who succumbed to sudden cardiovascular accident. Pathological examination revealed SARS-CoV-2-viruses remaining in pneumocytes and virus-caused pathological changes in the lungs. Our study provided new insights into SARS-CoV-2 pathogenesis and might facilitate the improvement of clinical guideline for virus containment and disease management.

A 78-year-old woman was admitted to hospital on January 27, 2020, due to falling-resulted trauma. This patient reported that she had been exposed to a COVID-19 patient on January 25th. Since January 29th, the patient showed pneumonia symptoms (Supplementary information, Fig. S1a). On Feburary 2nd, the patient was confirmed as SARS-CoV-2 positive by nasopharyngeal swab—PCR test followed by treatment (Supplementary information, Fig. S1a). On Feburary 3rd, chest scan by computerized tomography (CT) showed multiple patchy shadows in both lungs, implying pulmonary infection (Supplementary information, Fig. S1b). From Feburary 8th to 10th, three consecutive PCR tests on nasopharyngeal swab samples indicated SARS-CoV-2 negative (Supplementary information, Fig. S1a). From Feburary 11th to 13th, the patient’s condition was significantly improved, and CT examination showed absorption of pulmonary exudation (Supplementary information, Fig. S1a, b). Accordingly, the patient was ready for discharge. On Feburary 14th, however, this patient fell suddenly into fatal condition with cardiac arrest, and died unfortunately. Clinical laboratory test information was summarized in Supplementary information, Table S1, which revealed that the patient had lymphopenia, a frequent symptom for COVID-19 patients.

Regardless of the negative detection of SARS-CoV-2 virus nucleic acid from nasopharyngeal swabs, we sought to determine whether there were SARS-CoV-2 viruses remaining in the patient. We performed digital PCR on tissue sections from the lung, liver, heart, intestine, and skin, and unexpectedly found positive SARS-CoV-2 virus nucleic acid only in the lung, but not other tissues (Supplementary information, Fig. S2). Consistently, electron microscopic observation showed clear coronavirus particles in both bronchiolar epithelial cells marked by cilia and type II alveolar epithelial cells (type II AE) featured with lamellar body. The diameters of virus particles were 70–100 nm (Fig. 1a, b). Furthermore, we conducted immunohistochemical (IHC) staining by using monoclonal antibody against SARS-CoV-2 nucleocapsid, and confirmed SARS-CoV-2 viruses existed in the lung tissue (Fig. 1c). Neither coronavirus particles nor SARS-CoV-2 nucleocapsid were detected in the liver, heart, intestine, skin, and bone marrow. These results highlight the remaining of SARS-CoV-2 in the lung of discharged COVID-19 patient.

**Fig. 1:**
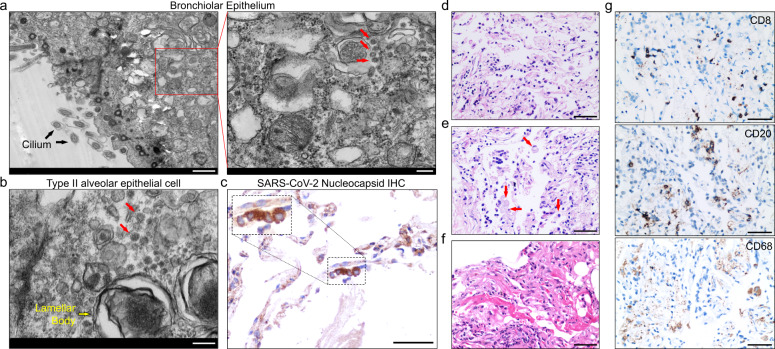
Pathological observation of the lung tissues. **a** Electron microscopic examination on a single pulmonary bronchiolar epithelial cell. Black arrows in left panel indicate organelle in pulmonary epithelial cell. Red arrows in right panel label virus particles. Scale bar: 1 μm in left panel and 200 nm in right panel. **b** Electron microscopic examination on a single type II alveolar epithelial cell. Yellow arrow indicates organelle in pulmonary epithelial cell. Red arrows label virus particles. Scale bar: 200 nm. **c** Immunohistochemical (IHC) staining of SARS-CoV-2 nucleoprotein (N) in pulmonary tissue with monoclonal anti-nucleoprotein antibody. The inset represents magnification of the selected area. Dark brown signals indicate positive staining for SARS-CoV-2 nucleoprotein and nuclei are counterstained with hematoxylin. Scale bar: 50 μm. **d** H&E staining shows desquamated and enlarged epithelial cells. Scale bar: 50 μm. **e** H&E staining shows exudative monocytes/macrophages in alveoli. Red arrows show typical macrophages in alveoli. Scale bar: 50 μm. **f** H&E staining shows formation of hyaline membranes. Scale bar: 50 μm. **g** IHC staining indicates lung-infiltrated immune cells: CD68^+^ macrophages, CD20^+^ B cells, and CD8^+^ T cells. Scale bar: 50 μm.

Histopathological examination of the samples from pulmonary biopsy showed predominant diffuse alveolar damage, exemplified by the extensive desquamation of proliferative type II AE, exudative monocytes and macrophages. Some of alveolar walls were partially lined by low columnar type II AE and covered by the formation of hyaline membranes in alveolar space. Thickening of alveolar septa with scattered interstitial inflammatory infiltration and hyaline thrombus in microvessels, but no pulmonary edema was found (Fig. 1d–f). There were also chronic respiratory disease-associated changes in the lung tissues. To further delineate the cell types of infiltrated immune cells in alveolar space and septa, we performed IHC staining and found that they were predominantly infiltrating CD68^+^ macrophages, CD20^+^ B cells, and CD8^+^ T cells (Fig. 1g). CD4^+^ T and CD38^+^ plasma cells were barely detectable (data not shown).

Pathological features of COVID-19,^4^ especially in the pulmonary tissues of mild and recovering patients, remain largely unknown. In this study, we conducted postmortem study in an aged patient with mild COVID-19 pneumonia and found pathological changes of the lungs caused by SARS-CoV-2 infection. Histologically, we observed that the patient’s lung was predominated with diffuse alveolar damages, including disrupt of alveolar septa, proliferation and desquamation of type II AE, exudation of fibrin, monocytes and macrophages, and formation of hyaline membrane. These pulmonary pathologic features were consistent with those seen in SARS and Middle Eastern Respiratory Syndrome (MERS),^5–9^ highlighting that the successful methodology in managing SARS and MERS could be referred to COVID-2019 patients. By using comprehensive means including electron microscopy and IHC staining, we revealed remaining of SARS-CoV-2 in the lung from the ready-for-discharge patient, which raises a possibility that nasopharyngeal swab negative result might not reflect the virus in lung tissue. In addition, our work provided the first pathological evidence for residual virus in the lung for a patient with virus negative by nasopharyngeal swab—PCR test for consecutive three times. Therefore, PCR detection of SARS-CoV-2 nucleic acid on broncho-alveolar lavage fluid, extension of quarantine time, and the timely follow-up medical examination on discharged patients, especially aged ones with underlying diseases, were strongly recommended for discharged patients.

## Supplementary information

Supplementary information
