# Supplementary material for: Pathological evidence for residual SARS-CoV-2 in pulmonary tissues of a ready-for-discharge patient
Source: Cell Res. 2020 Apr 28;30(6):541–3. doi: 10.1038/s41422-020-0318-5 (PMC7186763; doi:10.1038/s41422-020-0318-5)
Supplement: Supplementary file 1 — Supplementary information [file 41422_2020_318_MOESM1_ESM.pdf]

Supplementary Information, Figures

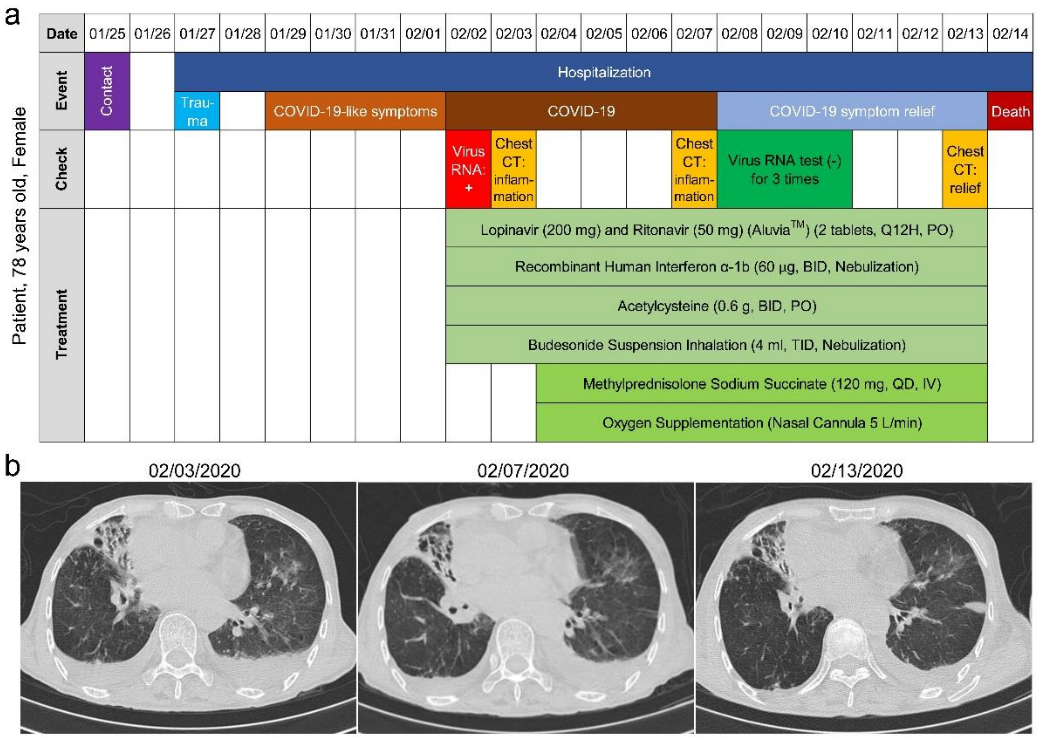

Supplementary information, Fig. S1. Clinical description and test for the patient. a) Outline of disease course and medical treatment for the patient. **b)** Three chest CT scan images of the patient.

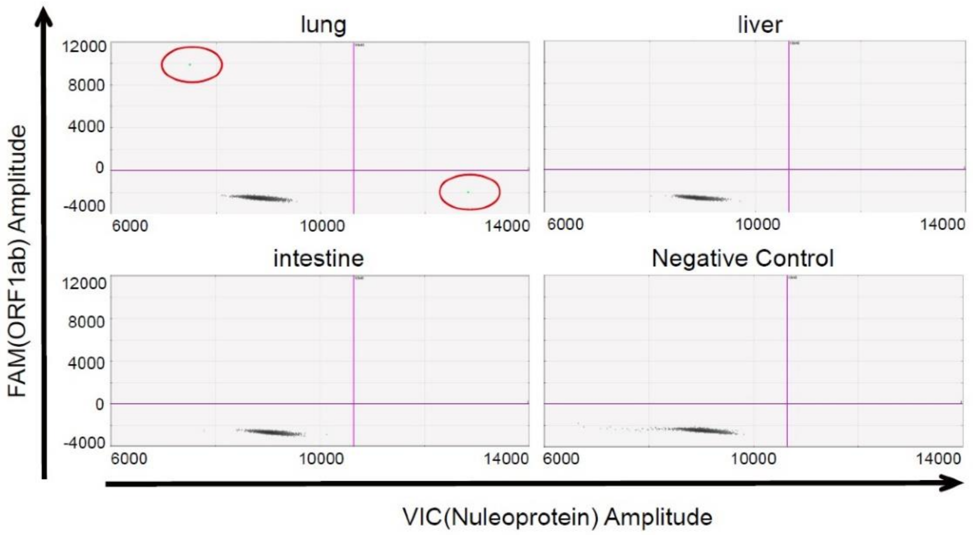

Supplementary information, Fig. S1. Digital PCR examination for virus from multiple tissue biopsies. Red oval indicates positive signal of virus RNA in lung from the patient died of COVID-19.

Supplementary Information, Tables

Supplementary information, Table S1. Clinical laboratory tests of the patient.

Hospitalization Day	Feb 2	3	4	5	6	7	8	9	10	11	12	Reference range
Oxygen saturation (%)	97	99.4	99	100	99	99	99	99	99	96	99	> 95
Body temperature (°C)	< 37.0	36.7	< 37.0							36.3	36.6	< 37.0
WBC count (×10^9/L)	5.1	6	5.8	11.9↑	16.2↑	11.5↑	12.7↑	9.3	9.3	10.8↑	14.9↑	3.97-9.15
Neutrophil percentage (%)	80.6↑	79.3↑	89.2↑	87.4↑	92.1↑	91.8↑	86.9↑	79↑	79↑	74.9↑	85.9↑	50-70
Lymphocyte percentage (%)	11.7↓	11.5↓								14.5↓		20-40
RBC count (×10^12/L)	3.31↓	2.7↓										4.09-5.74
Hemoglobin (g/L)	86↓	70↓	75↓	72↓	98↓	111↓		128↓			68↓	131-172
Platelet count (×10^9/L)	138↓	156									123↓	150-400
Total protein (g/L)	58.1↓					54.2↓	50.3↓		54.9↓	50.9↓	45.2↓	60-83
Albumin (g/L)	32.1↓					32↓	31.1↓		32.5↓	31.3↓	30↓	35-55
Sodium (mmol/L)	140	145			131↓		139			140	133↓	136-145
Potassium (mmol/L)	3.38↓	3.4↓			5.05	3.31↓	4.05		4.32	5.33↑		3.5-5.2
Chloride (mmol/L)	100	116↑						98				93-108

Supplementary information, Table S2. Antibody information used for immunohistochemistry.

Primary antibody	Company	Catalog #	Host species	Dilution	Antigen retrieval
CD68	MXB Biotechnologies	KIT-0026	Mouse	Ready to use	EDTA (pH 9.0)
CD8	MXB Biotechnologies	RMA-0514	Rabbit	Ready to use	EDTA (pH 9.0)
CD20	MXB Biotechnologies	KIT-0001	Mouse	Ready to use	EDTA (pH 9.0)
SARS-CoV-2 Nucleoprotein	Sino Biological	40143-R019	Rabbit	1:800	Citrate (pH 6.0)
CD4	MXB Biotechnologies	RMA-0620	Rabbit	Ready to use	EDTA (pH 9.0)
CD38	MXB Biotechnologies	MAB-0755	Mouse	Ready to use	EDTA (pH 9.0)

Supplementary information, Table S3. The primers and probes used in RT-PCR.

	Target 1 (open reading frame 1ab, ORF1ab)	Target 2 (nucleoprotein, N)
Forward	5'-CCCTGTGGGTTTTACACTTAA-3'	5'-GGGGAACTTCTCCTGCTAGAAT-3'
Reverse	5'-ACGATTGTGCATCAGCTGA-3'	5'-CAGACATTTTGCTCTCAAGCTG-3'
Probe	5'-FAM-CCGTCTGCGGTATGTGGAAAGGTTATGG-BHQ1-3'	5'-FAM-TTGCTGCTGCTTGACAGATT-TAMRA-3'

Supplementary information, Materials and Methods

Specimen collection

This study was conducted in accordance with regulations issued by the National Health Commission of China and the Helsinki Declaration. With written consent from patient family, specimens of the lung, liver, skin, intestine, bone marrow tissue, and heart by needle biopsy were collected, and then divided into two pieces. One was fixed in 4% neutral formaldehyde to make frozen tissue blocks or formalin-fixed paraffin-embedded (FFPE) tissue blocks, and another one was fixed in 2.5% glutaraldehyde (4 °C) for transmission electron microscopy.

Histochemical and immunohistochemical (IHC) staining

The FFPE tissue blocks were cut into 3 µm-thick serial sections. The sections were stained with hematoxylin and eosin (H&E) histochemical staining. Immunohistochemical staining was performed on the sections from each organ samples. Standard procedures of streptavidin peroxidase method were applied as previously described [1]. Heat-induced antigen epitope retrieval in EDTA (pH: 9.0) or citrate buffer (pH: 6.0) was applied for optimal detection of antigens on FFPE sections. Sections were incubated overnight at 4°C with primary antibodies against SARS-CoV-2 Nucleocapsid (N), CD4, CD8, CD20, CD31, CD38, and CD68 **(Table S2)**. Staining were visualized by Dako REAL™ EnVision™ Detection System, Peroxidase/DAB+, Rabbit/Mouse (K5007, Dako) followed by counterstaining with hematoxylin. Images were captured using a digital camera (DP73, Olympus) under a light microscope (BX53, Olympus). The diluent without primary antibodies was used as negative control.

Transmission electron microscopy (TEM)

TEM was performed under a routine procedure [2]. In brief, specimens (approximately 1mm×1mm×1mm in size) from each organ were fixed in 2.5% glutaraldehyde in 0.1 M phosphoric buffer (pH: 7.4) for 24 hours, post-fixed with 1% osmium tetroxide, dehydrated with gradient alcohol, and embedded using Eponate 12™ Kit with DMP-30 (18010, TED PELLA Inc.). The blocks were cut into 100 nm-thick sections, then double-stained with uranium acetate and lead citramalic acid, separately. The sections were observed under a Hitachi HT7700 transmission electron microscope.

Quantitative reverse-transcriptase polymerase chain reaction (qRT-PCR) and digital droplet PCR (ddPCR)

Total RNA of FFPE tissue samples from each organ were extracted using a FFPE RNA Kit (ADx-FF04, Amoy Diagnostics Co., Ltd, China). The nucleic acids of SARS-Cov-2 were detected by real-time RT-PCR method with a SARS-Cov-2 Nucleic Acid Detection Kit (8.0131901X024E, Amoy Diagnostics Co., Ltd) according to the manufacturer's instructions. Digital droplet PCR (ddPCR) assays were performed on QX200 AutoDG Droplet Digital PCR system (Bio-Rad) with One-Step RT-ddPCR Advanced Kit (186-4021, BIO-RAD Co., Ltd) according to the manufacturer's instructions. The sequences of primers and probes of SARS-Cov-2 were obtained from National Institute for Viral Disease Control and Prevention (http://nmdc.cn/#/nCoV) which were listed in **Table S3**.

References

1 Ding, Y. et al. *J. Pathol.* **203**, 622-630 (2004).
2 Goldsmith, C. S. et al. *Emerg. Infect. Dis.* **10**, 320-326 (2004).
